# Supplementary material for: IGFBP5 is an ROR1 ligand promoting glioblastoma invasion via ROR1/HER2-CREB signaling axis
Source: Nat Commun. 2023 Mar 22;14:1578. doi: 10.1038/s41467-023-37306-1 (PMC10033905; doi:10.1038/s41467-023-37306-1)
Supplement: Supplementary file 1 — Supplementary Information [file 41467_2023_37306_MOESM1_ESM.pdf]

Supplementary Information

IGFBP5 is an ROR1 ligand promoting glioblastoma invasion via ROR1/HER2-CREB signaling axis

Weiwei Lin[1,2,3,4,8], Rui Niu[1,8], Seong-Min Park[2,5,8], Yan Zou[1,6,8], Sung Soo Kim[2,8], Xue Xia[1], Songge Xing[1], Qingshan Yang[1], Xinhong Sun[1], Zheng Yuan[1], Shuchang Zhou[1], Dongya Zhang[1], Hyung Joon Kwon[7], Saewhan Park[2], Chan Il Kim[2], Harim Koo[2], Yang Liu[1], Haigang Wu[1], Meng Zheng[1], Heon Yoo[2,3], Bingyang Shi[1,6*], Jong Bae Park[1,2,3*] & Jinlong Yin[1,2*]

[1]Henan-Macquarie University Joint Centre for Biomedical Innovation, School of Life Sciences, Henan University, Kaifeng, Henan 475004, China. [2]Department of Cancer Biomedical Science, Graduate School of Cancer Science and Policy, National Cancer Center, Goyang, Gyeonggi 10408, Republic of Korea. [3]Research Institute, National Cancer Center, Goyang, Gyeonggi 10408, Republic of Korea. [4]Department of Life Science, Ewha Womans University, Seoul 03760, Republic of Korea. [5]Personalized Genomic Medicine Research Center, KRIBB, Daejeon 34141, Republic of Korea. [6]Centre for Motor Neuron Disease Research, Macquarie Medical School, Faculty of Medicine & Health Sciences, Macquarie University, Sydney, NSW 2109, Australia. [7]Department of Cancer Control and Population Health, Graduate School of Cancer Science and Policy, National Cancer Center, Goyang, Gyeonggi 10408, Republic of Korea. [8]These authors contributed equally to this work: Weiwei Lin, Rui Niu, Seong-Min Park, Yan Zou, Sung Soo Kim. [*]e-mail: jlyin@henu.edu.cn; jbp@ncc.re.kr; bs@henu.edu.cn.

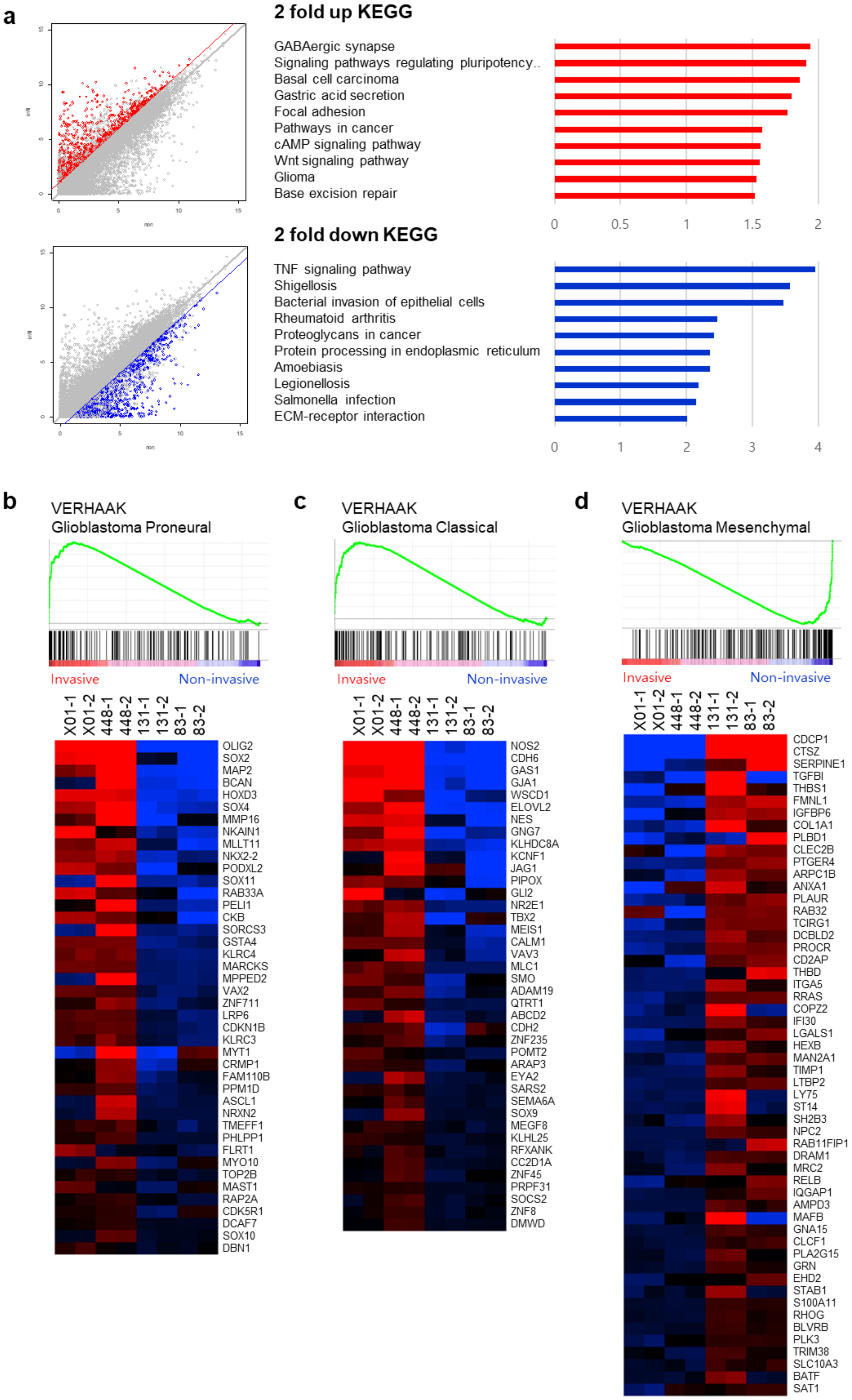
a
2 fold up KEGG
GABAergic synapse
Signaling pathways regulating pluripotency..
Basal cell carcinoma
Gastric acid secretion
Focal adhesion
Pathways in cancer
cAMP signaling pathway
Wnt signaling pathway
Glioma
Base excision repair
0
0.5
1
1.5
2
2 fold down KEGG
TNF signaling pathway
Shigellosis
Bacterial invasion of epithelial cells
Rheumatoid arthritis
Proteoglycans in cancer
Protein processing in endoplasmic reticulum
Amoebiasis
Legionellosis
Salmonella infection
ECM-receptor interaction
0
1
2
3
4
b
VERHAAK
Glioblastoma Proneural
Invasive
Non-invasive
X01-1
X01-2
448-1
448-2
131-1
131-2
83-1
83-2
OLIG2
SOX2
MAP2
BCAN
HOXD3
SOX4
MMP16
NKAIN1
MLLT11
NKX2-2
PODXL2
SOX11
RAB33A
PELI1
CKB
SORCS3
GSTA4
KLRC4
MARCKS
MPPED2
VAX2
ZNF711
LRP6
CDKN1B
KLRC3
MYT1
CRMP1
FAM110B
PPM1D
ASCL1
NRXN2
TMEFF1
PHLPP1
FLRT1
MYO10
TOP2B
MAST1
RAP2A
CDK5R1
DCAF7
SOX10
DBN1
c
VERHAAK
Glioblastoma Classical
Invasive
Non-invasive
X01-1
X01-2
448-1
448-2
131-1
131-2
83-1
83-2
NOS2
CDH6
GAS1
GJA1
WSCD1
ELOVL2
NES
GNG7
KLHDC8A
KCNF1
JAG1
PIPOX
GLI2
NR2E1
TBX2
MEIS1
CALM1
VAV3
MLC1
SMO
ADAM19
QTRT1
ABCD2
CDH2
ZNF235
POMT2
ARAP3
EYA2
SARS2
SEMA6A
SOX9
MEGF8
KLHL25
RFXANK
CC2D1A
ZNF45
PRPF31
SOCS2
ZNF8
DMWD
d
VERHAAK
Glioblastoma Mesenchymal
Invasive
Non-invasive
X01-1
X01-2
448-1
448-2
131-1
131-2
83-1
83-2
CDCP1
CTSZ
SERPINE1
TGFBI
THBS1
FMNL1
IGFBP6
COL1A1
PLBD1
CLEC2B
PTGER4
ARPC1B
ANXA1
PLAUR
RAB32
TCIRG1
DCBLD2
PROCR
CD2AP
THBD
ITGA5
RRAS
COPZ2
IFI30
LGALS1
HEXB
MAN2A1
TIMP1
LTBP2
LY75
ST14
SH2B3
NPC2
RAB11FIP1
DRAM1
MRC2
RELB
IQGAP1
AMPD3
MAFB
GNA15
CLCF1
PLA2G15
GRN
EHD2
STAB1
S100A11
RHOG
BLVRB
PLK3
TRIM38
SLC10A3
BATF
SAT1

Supplementary Fig. 1

Different expression patterns and pathways in invasive and non-invasive GSCs (RNA-seq). a, KEGG pathway analysis of invasive and non-invasive GSCs. **b-d,** Gene set enrichment analysis (GSEA) plots of invasive and non-invasive of GSCs focusing on the GBM proneural signature **(b),** GBM classical signature **(c),** and GBM mesenchymal signature **(d).**

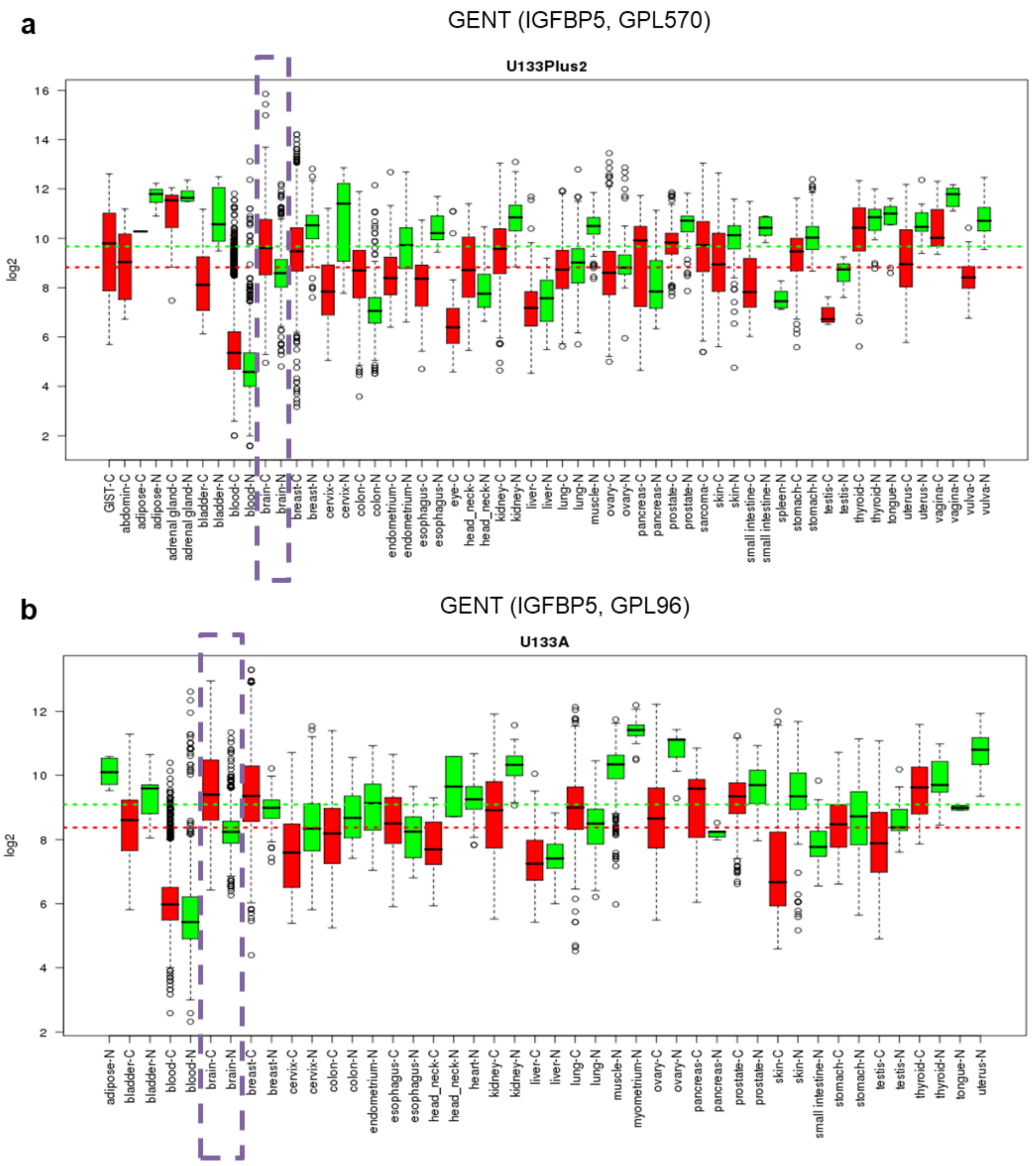

Supplementary Fig. 2

IGFBP5 expression in normal and tumor tissues of various cancer types (GENT database). **a,** Analysis of IGFBP5 expression in normal and cancer tissues based on the GeneChip Human Genome U133 Plus 2.0 Array. **b,** Analysis of IGFBP5 expression in normal and cancer tissues based on the Affymetrix Human Genome U133A Array. Both **a** and **b** originate from the GENT database (http://medical-genome.kribb.re.kr/GENT/), which presents over 3,4000 patient samples. Boxes are presentative as mean ± standard deviation (SD), bars show as mean ±2 * SD, bold lines in box are median, and dots are outlier samples.

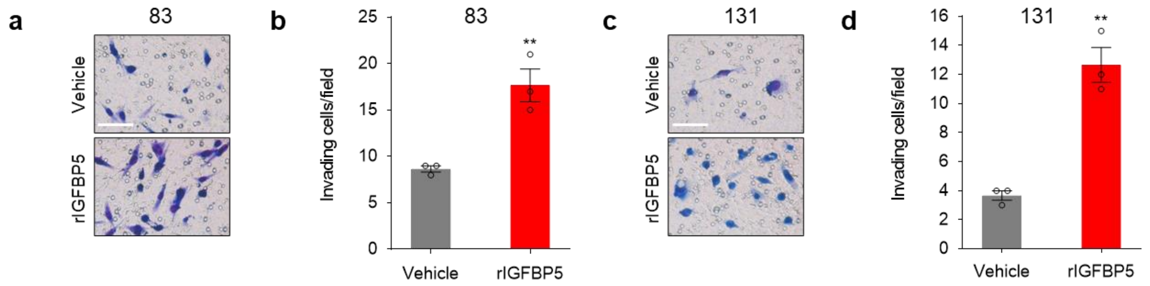

Supplementary Fig. 3

Recombinant IGFBP5 promotes GSC invasion *in vitro*. a-b, Invasion assays with 83 GSCs treated with 100 ng/ml rIGFBP5 or vehicle control for 6 h. Images **(a)** taken after 6 h of invasion are representative of three independent experiments (scale bar, 100 µm; n = 3), and the graph **(b)** shows the mean number of invasive cells ± SEM (n = 3 independent experiments), two-tailed Student's *t*-test ($^{**}P = 0.007$). **c-d,** Invasion assays using 131 GSCs treated with 100 ng/ml rIGFBP5 or vehicle control for 6 h. Images **(c)** taken after 6 h of invasion are representative of three independent experiments (scale bar, 100 µm; n = 3), and the graph **(d)** shows the mean number of invasive cells ± SEM (n = 3 independent experiments), two-tailed Student's *t*-test ($^{**}P = 0.002$). Source data are provided as the Source Data file.

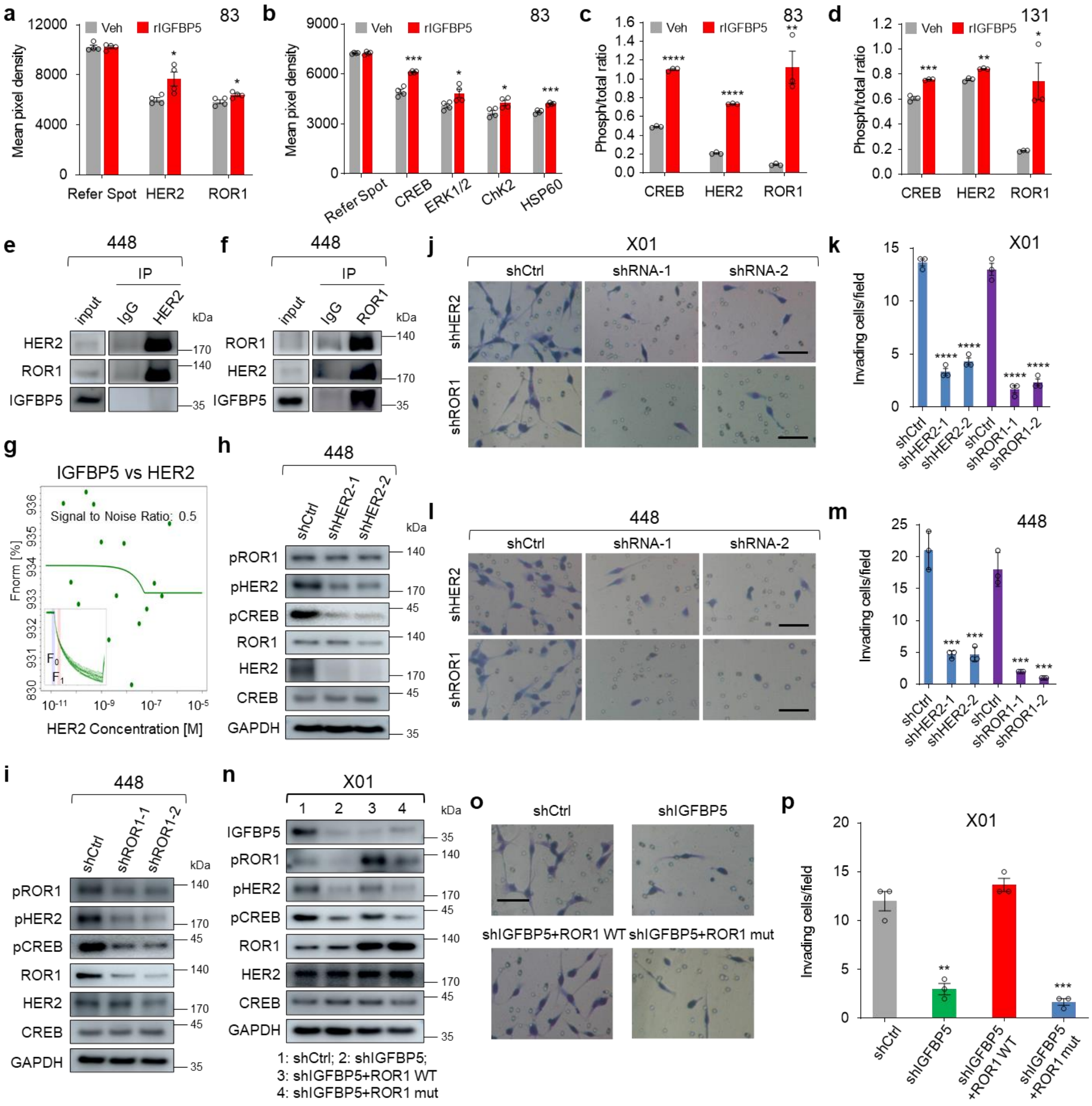
a
83
Veh
rIGFBP5
Mean pixel density
12000
8000
4000
0
Refer Spot
HER2
ROR1
b
83
9000
6000
3000
0
Refer Spot
CREB
ERK1/2
ChK2
HSP60
c
83
Phosph/total ratio
1.6
1.4
1.2
1.0
0.8
0.6
0.4
0.2
0.0
CREB
HER2
ROR1
d
131
1.2
1.0
0.8
0.6
0.4
0.2
0.0
e
448
IP
input
IgG
HER2
kDa
HER2
ROR1
IGFBP5
170
140
35
f
448
IP
input
IgG
ROR1
kDa
ROR1
HER2
IGFBP5
140
170
35
g
IGFBP5 vs HER2
Signal to Noise Ratio: 0.5
Fnorm [%]
HER2 Concentration [M]
h
448
shCtrl
shHER2-1
shHER2-2
kDa
pROR1
pHER2
pCREB
ROR1
HER2
CREB
GAPDH
140
170
45
35
i
448
shCtrl
shROR1-1
shROR1-2
j
X01
shCtrl
shRNA-1
shRNA-2
shHER2
shROR1
k
X01
Invading cells/field
15
10
5
0
shCtrl
shHER2-1
shHER2-2
shROR1-1
shROR1-2
l
448
m
448
25
20
n
X01
1
2
3
4
IGFBP5
1: shCtrl; 2: shIGFBP5;
3: shIGFBP5+ROR1 WT
4: shIGFBP5+ROR1 mut
o
shCtrl
shIGFBP5
shIGFBP5+ROR1 WT
shIGFBP5+ROR1 mut
p
X01
shIGFBP5
+ROR1 WT
+ROR1 mut

Supplementary Fig. 4

HER2 and ROR1 expression regulate GSCs invasion. a-b, Quantitative analysis of the mean pixel density between vehicle control (Veh) and rIGFBP5 (100ng/ml recombinant IGFBP5 protein treated 6 h) for RTK array from Fig. 4a **(a)**, for Kinase array from Fig. 4b **(b)** in 83 GSCs by using ImageJ software. Data are presentative as mean ± SEM of 4 independent experiments (n = 4), two-tailed Student's *t*-test (left to right, (**a**) $^{*}P = 0.03$, $^{*}P = 0.03$; (**b**) $^{***}P = 0.0002$, $^{*}P = 0.03$, $^{*}P = 0.03$, $^{***}P = 0.0003$). Refer Spots were used as loading controls. **c-d,** Bar graph shows the optical density ratio of pCREB/CREB, pHER2/HER2, and pROR1/ROR1 from Fig. 4c 83 **(c)** and 131 **(d)** using ImageJ software, Data are presentative as mean ± SEM of 3 independent experiments (n = 3), two-tailed Student's *t*-test (left to right, (**c**) $^{****}P = 0.0000007$, $^{****}P = 0.0000002$, $^{**}P = 0.004$; (**d**) $^{***}P = 0.0003$, $^{**}P = 0.002$, $^{*}P = 0.02$). **e-f,** Co-IP of 448 GSCs with antibodies targeting HER2 **(e),** ROR1 **(f)** or normal IgG. **g,** *In vitro*-binding affinity between IGFBP5 and HER2 was tested by MST assay. **h-i,** IB analysis of pROR1, pHER2, pCREB, ROR1, HER2, and CREB in 448 GSCs infected with shCtrl, shRNA-1 and shRNA-2 of HER2 **(h),** or ROR1 **(i)** lentivirus. GAPDH was used as a loading control. **j-m,** Invasion assays of X01 **(j, k)** and 448 **(l, m)** GSCs infected with shCtrl, shRNA-1 and shRNA-2 of HER2, or ROR1 lentivirus. Images **(j, l)** taken after 24 h of invasion are representative of three independent experiments (scale bar, 100 μm; n = 3), and the graph **(k, m)** shows the mean number of the invasive cells ± SEM, two-tailed Student's *t*-test (left to right, (**k**) $^{****}P = 0.00003$, $^{****}P = 0.00004$, $^{****}P = 0.00007$, $^{****}P = 0.00009$; (**m**) $^{***}P = 0.0008$, $^{***}P = 0.0009$, $^{***}P = 0.0005$, $^{***}P = 0.0004$). **n,** IB analysis of pROR1, pHER2, pCREB, ROR1, HER2, and CREB in X01 GSCs infected with shCtrl, shIGFBP5-1 lentivirus, and then infected with vector control, ROR1-WT, or ROR1-mut lentivirus. GAPDH was used as a loading control. **o-p,** Invasion assays of X01 GSCs infected with shCtrl, shIGFBP5-1 lentivirus, and then infected with vector control, ROR1-WT, or ROR1-mut lentivirus. Images **(o)** taken after 24 h of invasion are representative of three independent experiments (scale bar, 100 μm; n = 3), and the graph **(p)** shows the mean number of the invasive cells ± SEM, two-tailed Student's *t*-test (left to right, $^{**}P = 0.001$, $^{ns}P = 0.24$, $^{***}P = 0.0006$). All the immunoblots were representative data from three independent experiments. Source data are provided as the Source Data file.

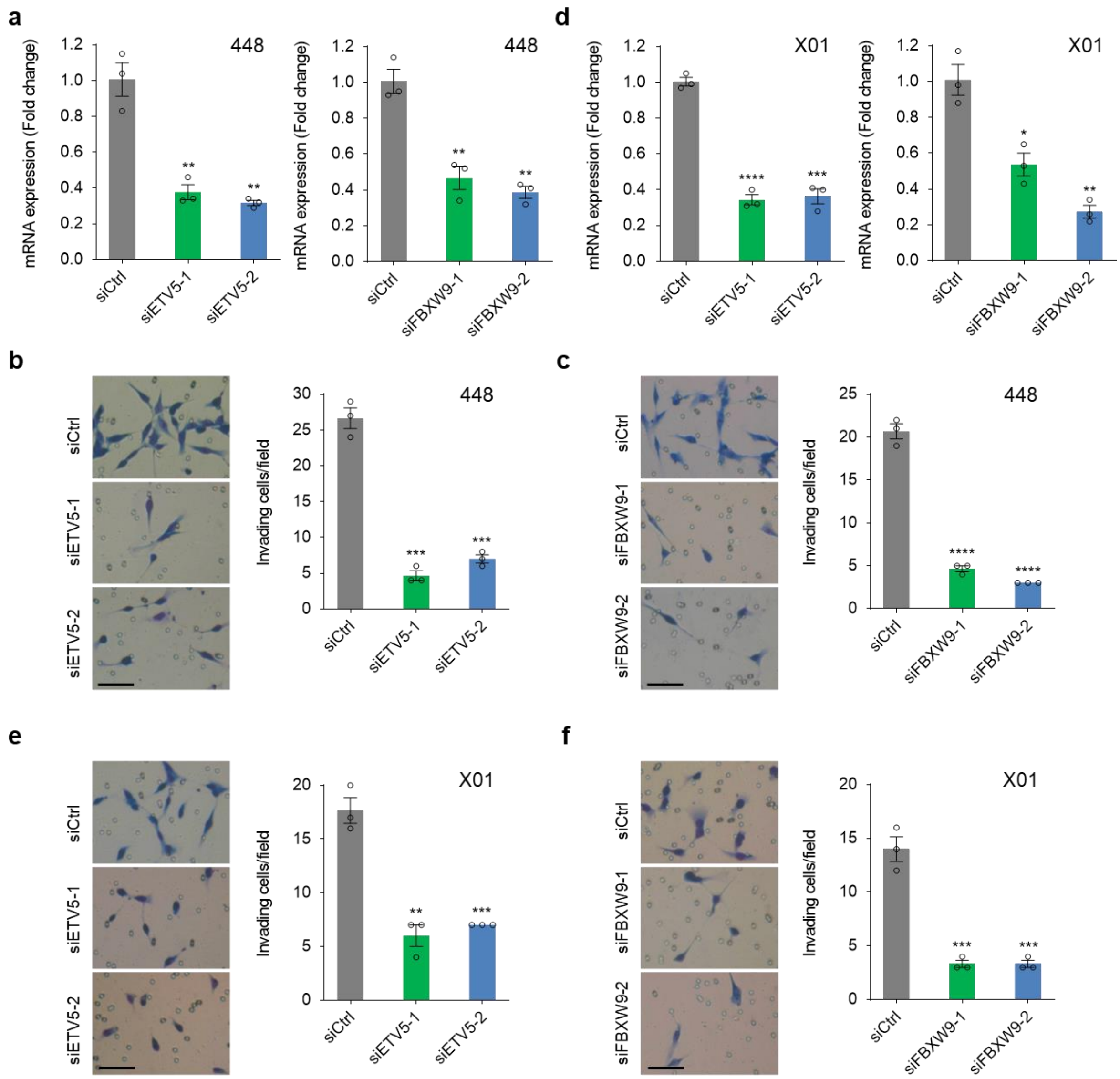
a
448
mRNA expression (Fold change)
1.2
1.0
0.8
0.6
0.4
0.2
0.0
siCtrl
siETV5-1
siETV5-2
siFBXW9-1
siFBXW9-2
d
X01
b
Invading cells/field
siCtrl
siETV5-1
siETV5-2
c
siFBXW9-1
siFBXW9-2
e
f

Supplementary Fig. 5

Inhibition of ETV5 and FBXW9 reduces GSCs invasion. a, RT-qPCR analysis of ETV5 (left) and FBXW9 (right) expression in 448 GSCs transfected with siRNAs of EVT5 (left) and FBXW9 (right). Data are presented as mean ± SEM (n = 3 independent experiments), two-tailed Student's *t*-test (left to right, $^{**}P = 0.004$, $^{**}P = 0.002$, $^{**}P = 0.004$, $^{**}P = 0.001$). **b-c,** Invasion assays of 448 GSCs transfected with siRNAs of ETV5 **(b)** and FBXW9 **(c)**. Images (left) taken after 24 h of invasion are representative of three independent experiments (scale bar, 100 µm; n = 3), and the graph (right) shows the mean number of invasive cells ± SEM, two-tailed Student's *t*-test (left to right, $^{***}P = 0.0002$, $^{***}P = 0.0002$; $^{****}P = 0.00007$, $^{****}P = 0.00004$). **d,** RT-qPCR analysis of ETV5 (left) and FBXW9 (right) expression in X01 GSCs transfected with siRNA of EVT5 (left) and FBXW9 (right). Data are presented as mean ± SEM (n = 3 independent experiments), two-tailed Student's *t*-test (left to right, $^{****}P = 0.00005$, $^{***}P = 0.0002$, $^{*}P = 0.01$, $^{**}P = 0.001$). **e-f,** Invasion assays of X01 GSCs transfected with siRNA of ETV5 **(e)** and FBXW9 **(f)**. Images (left) taken after 24 h of invasion are representative of three independent experiments (scale bar, 100 µm; n = 3), and the graph (right) shows the mean number of invasive cells ± SEM, two-tailed Student's *t*-test (left to right, $^{**}P = 0.002$, $^{***}P = 0.0009$; $^{***}P = 0.0009$, $^{***}P = 0.0009$). Source data are provided as the Source Data file.

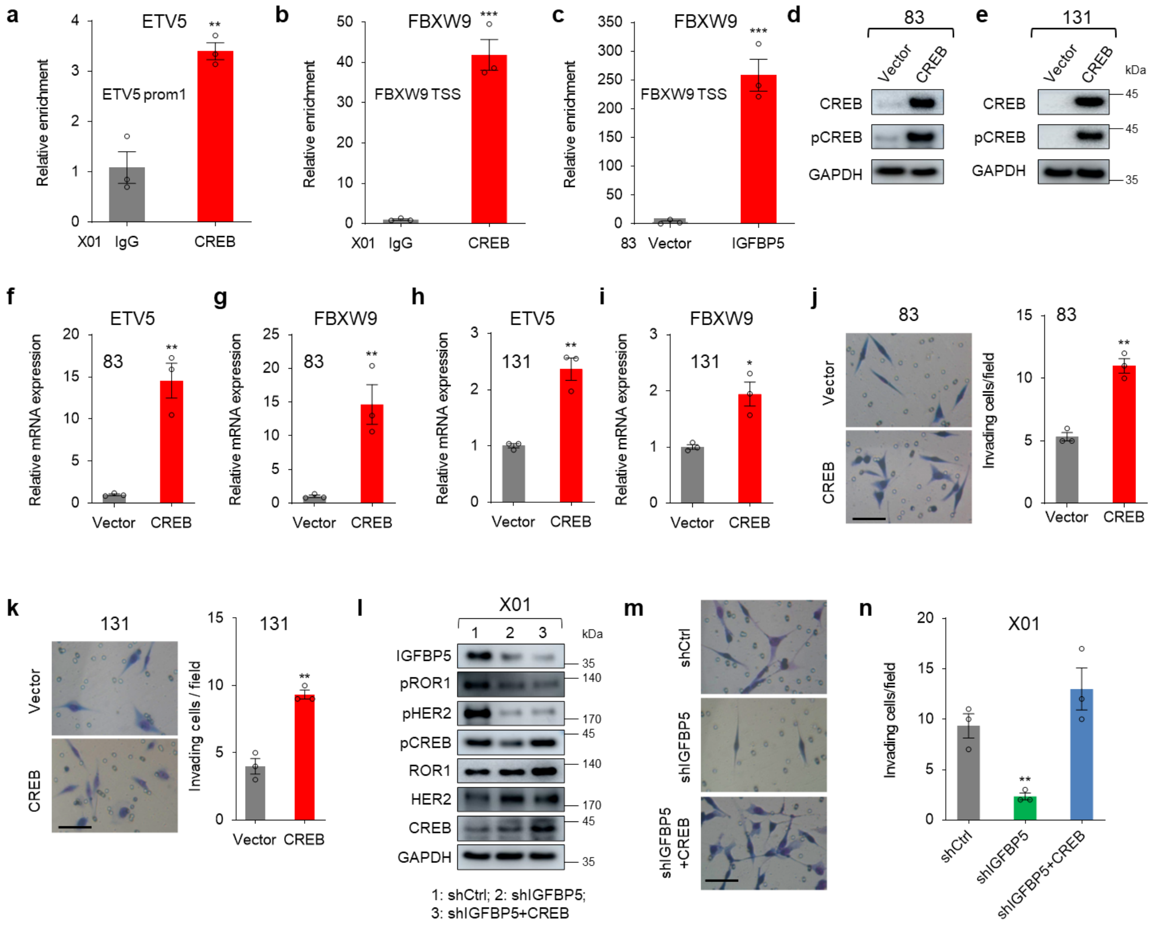
a
ETV5
ETV5 prom1
Relative enrichment
X01 IgG CREB
b
FBXW9
FBXW9 TSS
Relative enrichment
X01 IgG CREB
c
FBXW9
FBXW9 TSS
Relative enrichment
83 Vector IGFBP5
d
83
Vector CREB
CREB
pCREB
GAPDH
e
131
Vector CREB
kDa
CREB
pCREB
GAPDH
f
ETV5
83
Relative mRNA expression
Vector CREB
g
FBXW9
83
Relative mRNA expression
Vector CREB
h
ETV5
131
Relative mRNA expression
Vector CREB
i
FBXW9
131
Relative mRNA expression
Vector CREB
j
83
Vector
CREB
Invading cells/field
Vector CREB
k
131
Vector
CREB
Invading cells / field
Vector CREB
l
X01
1 2 3
kDa
IGFBP5
pROR1
pHER2
pCREB
ROR1
HER2
CREB
GAPDH
1: shCtrl; 2: shIGFBP5;
3: shIGFBP5+CREB
m
shCtrl
shIGFBP5
shIGFBP5 +CREB
n
X01
Invading cells/field
shCtrl
shIGFBP5
shIGFBP5+CREB

Supplementary Fig. 6

CREB directly activates the transcription of *ETV5* and *FBXW9*. a-c, ChIP-qPCR analysis of CREB binding to the *ETV5* promoter **(a)** and *FBXW9* TSS **(b)** in X01 GSCs, and *FBXW9* TSS **(c)** in 83 GSCs infected with vector control or IGFBP5 lentivirus. Data are presented as mean ± SEM (n = 3 independent experiments), two-tailed Student's *t*-test (left to right, $^{**}P$ = 0.003; $^{***}P$ = 0.0004; $^{***}P$ = 0.0008). **d-e,** IB analysis of pCREB, and CREB in 83 **(d)** and in 131 **(e)** GSCs infected with vector control, or CREB OE lentivirus. GAPDH was used as a loading control. **f-g,** RT-qPCR analysis of CREB targets ETV5 **(f)** and FBXW9 **(g)** expression in 83 GSCs infected with vector control, CREB OE lentivirus. Data are presented as mean ± SEM (n = 3 independent experiments), two-tailed Student's *t*-test (left to right, $^{**}P$ = 0.003; $^{**}P$ = 0.0098). **h-i,** RT-qPCR analysis of CREB targets ETV5 **(h)** and FBXW9 **(i)** expression in 131 GSCs infected with vector control, CREB OE lentivirus. Data are presented as mean ± SEM (n = 3 independent experiments), two-tailed Student's *t*-test (left to right, $^{**}P$ = 0.002; $^{*}P$ = 0.01). **j-k,** Invasion assays of 83 **(j)** and 131 **(k)** GSCs infected with vector control, or CREB OE lentivirus. Images (left) taken after 48 h of invasion are representative of three independent experiments (scale bar, 100 µm; n = 3), and the graph (right) shows the mean number of the invasive cells ± SEM, two-tailed Student's *t*-test (**j** to **k**, $^{**}P$ = 0.001, $^{**}P$ = 0.001). **l,** IB analysis of pROR1, pHER2, pCREB, ROR1, HER2, and CREB in X01 GSCs infected with shCtrl, or shIGFBP5-1 lentivirus, and then infected with vector control, CREB OE lentivirus. GAPDH was used as a loading control. **m-n,** Invasion assays of X01 GSCs infected with shCtrl, shIGFBP5-1 lentivirus, and then infected with vector control, CREB OE lentivirus. Images **(m)** taken after 24 h of invasion are representative of three independent experiments (scale bar, 100 µm; n = 3), and the graph **(n)** shows the mean number of the invasive cells ± SEM, two-tailed Student's *t*-test (left to right, $^{**}P$ = 0.005, ^{ns}P = 0.2). All the immunoblots were representative data from three independent experiments. Source data are provided as the Source Data file.

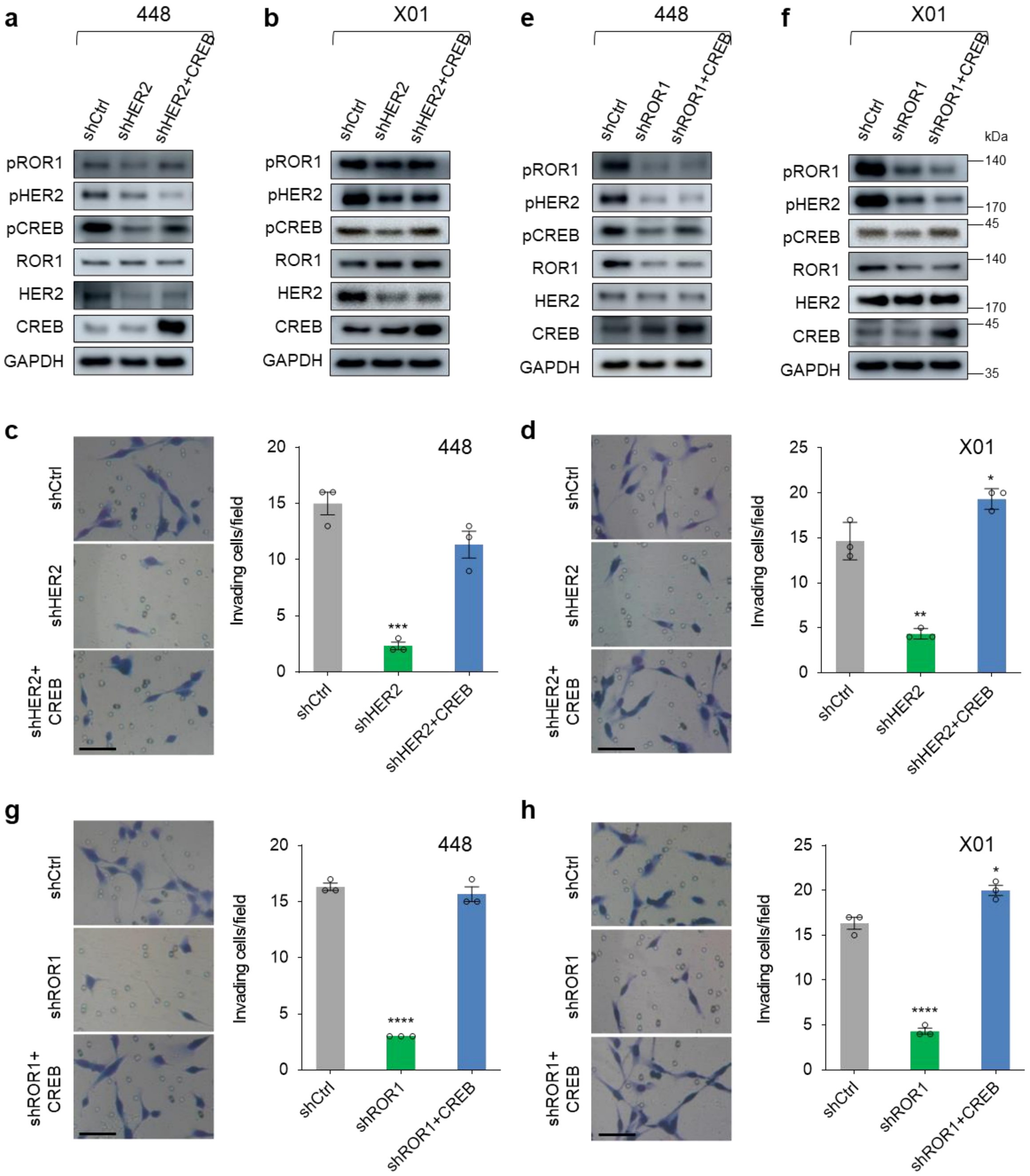
a
448
shCtrl
shHER2
shHER2+CREB
pROR1
pHER2
pCREB
ROR1
HER2
CREB
GAPDH
b
X01
e
shROR1
shROR1+CREB
f
kDa
140
170
45
140
170
45
35
c
shHER2+
CREB
Invading cells/field
0
5
10
15
20
***
d
25
**
*
g
****
h

Supplementary Fig. 7

Overexpression of CERB rescues HER2 or ROR1 knockdown-mediated repression of GSC invasion. a-b, IB analysis of pROR1, pHER2, pCREB, ROR1, HER2, and CREB in 448 **(a)** and X01 **(b)** GSCs infected with shCtrl, shHER2-1 lentivirus, and then infected with vector control, CREB OE lentivirus. GAPDH was used as a loading control. **c-d,** Invasion assays of 448 **(c)** and X01 **(d)** GSCs infected with shCtrl, shHER2-1 lentivirus, and then infected with vector control, CREB OE lentivirus. Images (left) taken after 24 h of invasion are representative of three independent experiments (scale bar, 100 µm; n = 3), and the graph (right) shows the mean number of the invasive cells ± SEM, two-tailed Student's *t*-test (left to right, (**c**) $^{***}P = 0.0003$, $^{ns}P = 0.08$; (**d**) $^{**}P = 0.001$, $^{*}P = 0.03$). **e-f,** IB analysis of pROR1, pHER2, pCREB, ROR1, HER2, and CREB in 448 **(e)** and X01 **(f)** GSCs infected with shCtrl, shROR1-1 lentivirus, and then infected with vector control, CREB OE lentivirus. GAPDH was used as a loading control. **g-h,** Invasion assays of 448 GSCs **(g)** and X01 **(h)** GSCs infected with shCtrl, shROR1-1 lentivirus, and then infected with vector control, CREB OE lentivirus. Images (left) taken after 24 h of invasion are representative of three independent experiments (scale bar, 100 µm; n = 3), and the graph (right) shows the mean number of invasive cells ± SEM, two-tailed Student's *t*-test (left to right, (**g**) $^{****}P = 0.000002$, $^{ns}P = 0.42$; (**h**) $^{****}P = 0.00009$, $^{*}P = 0.014$). All the immunoblots were representative data from three independent experiments. Source data are provided as the Source Data file.

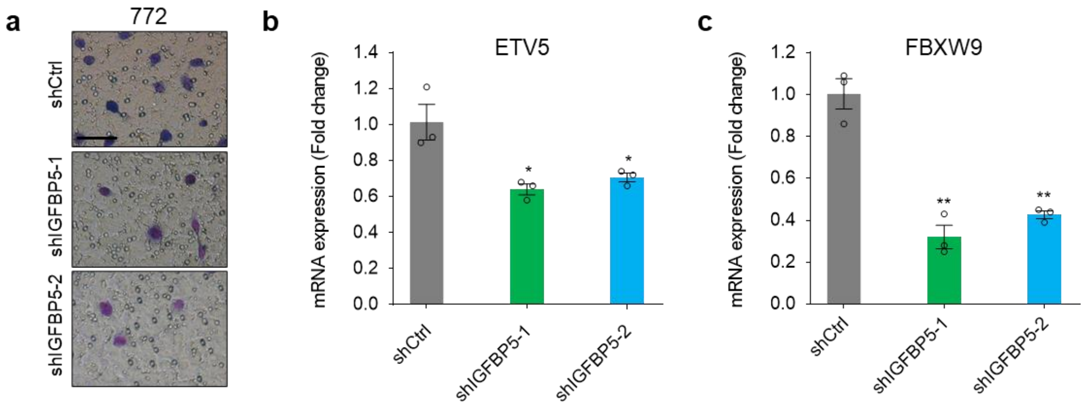

Supplementary Fig. 8

Regulation of GSC invasion by IGFBP5 in 772 GSCs. a, Invasion assays using patient-derived 772 GSCs infected with shCtrl, shIGFBP5-1, or shIGFBP5-2 lentivirus. Images taken after 48 h of invasion are representative of three independent experiments (scale bar, 100 μm; n = 3). **b-c,** RT-qPCR analysis of ETV5 **(b)** and FBXW9 **(c)** expression in patient-derived 772 GSCs infected with shCtrl, shIGFBP5-1, or shIGFBP5-2 lentivirus. Data are presented as mean ± SEM (n = 3 independent experiments), two-tailed Student's *t*-test (left to right, (**b**) $^{*}P = 0.02$, $^{*}P = 0.04$; (**c**) $^{**}P = 0.002$, $^{**}P = 0.001$). Source data are provided as the Source Data file.

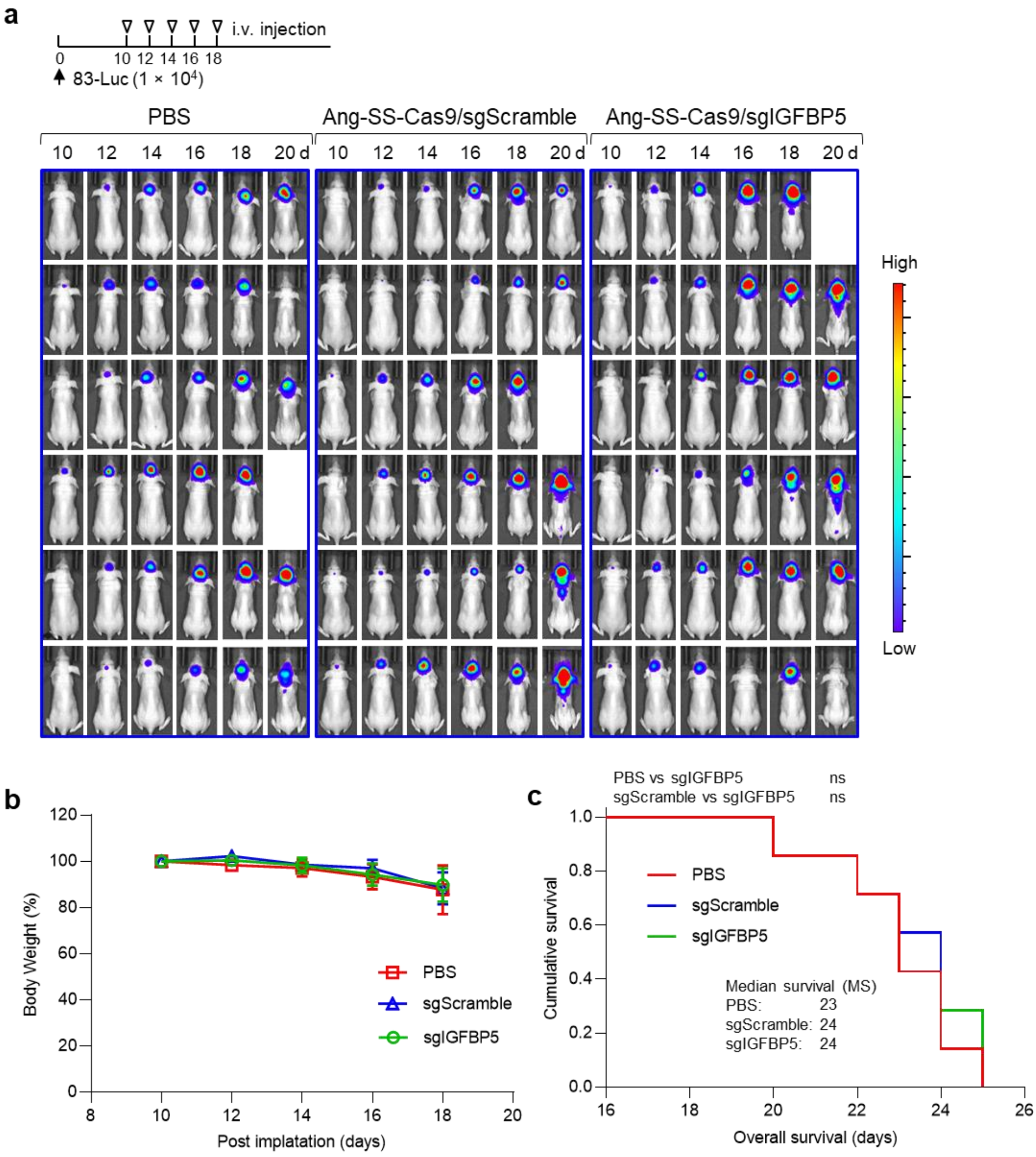
a
i.v. injection
0
10 12 14 16 18
83-Luc (1 × 10⁴)
PBS
Ang-SS-Cas9/sgScramble
Ang-SS-Cas9/sgIGFBP5
10 12 14 16 18 20 d
High
Low
b
Body Weight (%)
Post implatation (days)
PBS
sgScramble
sgIGFBP5
c
PBS vs sgIGFBP5 ns
sgScramble vs sgIGFBP5 ns
Cumulative survival
Overall survival (days)
PBS
sgScramble
sgIGFBP5
Median survival (MS)
PBS: 23
sgScramble: 24
sgIGFBP5: 24

Supplementary Fig. 9

Effect of Cas9/sgIGFBP5 nanocapsules on tumorigenesis in 83-Luc GSCs-bearing mice. **a,** Luminescence images of orthotopic 83-Luc GSCs-bearing mice following treatment with Ang-SS-Cas9/sgIGFBP5, Ang-SS-Cas9/sgScramble or PBS. Mice were intravenously injected at a dose of 1.5 mg Cas9 equiv./kg on day 10, 12, 14, 16, and 18 post tumor implantations. **b,** Body weight changes in mice following different treatments. Data is presentative as mean ± SEM (n = 7 in each group). **c,** Kaplan-Meier survival curves of mice implanted with 1×10^4 83-Luc GSCs and treated with Ang-SS-Cas9/sgIGFBP5, Ang-SS-Cas9/sgScramble or PBS (n = 7 in each group), $^{ns}P > 0.05$, log-rank test. Source data are provided as the Source Data file.

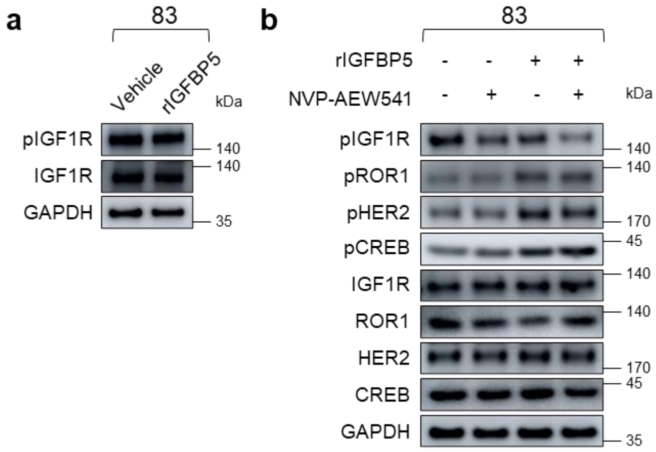

Supplementary Fig. 10

IGF1R does not regulate the IGFBP5 mediated signaling axis in GSCs. a, IB analysis of pIGF1R and IGF1R in 83 GSCs treated with rIGFBP5 (100 ng/ml) for 6 h. **b,** IB analysis of pIGF1R, pROR1, pHER2, pCREB, IGF1R, ROR1, HER2 and CREB in 83 GSCs with IGF1R blockade 3 h (10 μM NVP-AEW541) under IGFBP5 stimulation (100ng/ml rIGFBP5, 6 h). GAPDH was used as the loading control. All the immunoblots were representative data from three independent experiments. Source data are provided as the Source Data file.
