# Supplementary material for: IGFBP5 is an ROR1 ligand promoting glioblastoma invasion via ROR1/HER2-CREB signaling axis
Source: Nat Commun. 2023 Mar 22;14:1578. doi: 10.1038/s41467-023-37306-1 (PMC10033905; doi:10.1038/s41467-023-37306-1)
Supplement: Supplementary file 3 — Reporting Summary [file 41467_2023_37306_MOESM3_ESM.pdf]

nature portfolio

Corresponding author(s): Jinlong Yin

Last updated by author(s): Feb 8, 2023

Reporting Summary

Nature Portfolio wishes to improve the reproducibility of the work that we publish. This form provides structure for consistency and transparency in reporting. For further information on Nature Portfolio policies, see our Editorial Policies and the Editorial Policy Checklist.

Statistics

For all statistical analyses, confirm that the following items are present in the figure legend, table legend, main text, or Methods section.

n/a	Confirmed	
☐	☒	The exact sample size (n) for each experimental group/condition, given as a discrete number and unit of measurement
☐	☒	A statement on whether measurements were taken from distinct samples or whether the same sample was measured repeatedly
☐	☒	The statistical test(s) used AND whether they are one- or two-sided<br>*Only common tests should be described solely by name; describe more complex techniques in the Methods section.*
☐	☒	A description of all covariates tested
☐	☒	A description of any assumptions or corrections, such as tests of normality and adjustment for multiple comparisons
☐	☒	A full description of the statistical parameters including central tendency (e.g. means) or other basic estimates (e.g. regression coefficient) AND variation (e.g. standard deviation) or associated estimates of uncertainty (e.g. confidence intervals)
☐	☒	For null hypothesis testing, the test statistic (e.g. F, t, r) with confidence intervals, effect sizes, degrees of freedom and P value noted<br>*Give P values as exact values whenever suitable.*
☒	☐	For Bayesian analysis, information on the choice of priors and Markov chain Monte Carlo settings
☐	☒	For hierarchical and complex designs, identification of the appropriate level for tests and full reporting of outcomes
☐	☒	Estimates of effect sizes (e.g. Cohen's d, Pearson's r), indicating how they were calculated

Our web collection on statistics for biologists contains articles on many of the points above.

Software and code

Policy information about availability of computer code

Data collection	No software was used for data collection.
Data analysis	Microsoft Excel (2010), GraphPad Prism (ver7 and ver8), ImageJ (1.53), R (ver3.2.1), MeV (ver4.9.0), GSEA, Zetasizer Nano S, NTA 3.2 Dev Build 3.2.16, JEM1011, IVIS Lumina III, Caseviewe, Paravision (5.1).

For manuscripts utilizing custom algorithms or software that are central to the research but not yet described in published literature, software must be made available to editors and reviewers. We strongly encourage code deposition in a community repository (e.g. GitHub). See the Nature Portfolio guidelines for submitting code & software for further information.

Data

Policy information about availability of data

All manuscripts must include a data availability statement. This statement should provide the following information, where applicable:

- Accession codes, unique identifiers, or web links for publicly available datasets
- A description of any restrictions on data availability
- For clinical datasets or third party data, please ensure that the statement adheres to our policy

All IGFBP5 bioinformatic data was collected from International Cancer Genome Consortium (ICGC) data portal (http://dcc.icgc.org/) and GENT(http://medical-genome.kribb.re.kr/GENT/). RNA sequence data have been uploaded in Sequence Read Archive (SRA) with PRJNA732258 accession code. Accession codes for all datasets will be available without any restriction following Nature policy. All all the data all the databases/datasets used in the study along with appropriately

accessible links/accession-codes in the manuscript under the "Data availability" section in the manuscript.
The list of figures that have associated biological raw data are:
Figure 1 b, c, d, e, f, g, h;
Figure 2 a, b, d, f, g, h, i, j;
Figure 3 a, b, d, f, g, h, i, j;
Figure 4 a, b, c, d, e, f, g, h, i, j, k, i, m, n;
Figure 5 b, d, e, f, g, h, i, j, k, l;
Figure 6 a, b, c, d, e, f, g;
Figure 7 a, b, c, d, e, f, g, h, i, j, k, l, m;
Extended Data Figure 1, 3, 4, 5, 6, 7, 8, 9, 10.

Human research participants

Policy information about studies involving human research participants and Sex and Gender in Research.

Reporting on sex and gender	one 57-year-old male patient (772)
Population characteristics	Patient-derived tissues of brain tumor specimens diagnosed with glioblastoma were collected from one 57-year-old male patient (772) in National Cancer Center, South Korea.
Recruitment	Patient-derived tissues were obtained using excess material collected for clinical purposes from de-identified brain tumor specimens. The donor (glioblastoma patient) was anonymous. Progressive numbers were used to label specimens coded in order to preserve the confidentiality of the subjects.
Ethics oversight	This human research with these materials was covered IRB protocol NCC-2016-0181 in National Cancer Center, South Korea.

Note that full information on the approval of the study protocol must also be provided in the manuscript.

Field-specific reporting

Please select the one below that is the best fit for your research. If you are not sure, read the appropriate sections before making your selection.

☒ Life sciences ☐ Behavioural & social sciences ☐ Ecological, evolutionary & environmental sciences

For a reference copy of the document with all sections, see nature.com/documents/nr-reporting-summary-flat.pdf

Life sciences study design

All studies must disclose on these points even when the disclosure is negative.

Sample size	No statistical methods were used to predetermine sample size. All available samples passing the quality control were included.
Data exclusions	No data were excluded from analysis.
Replication	Three technical were performed and experiments were repeated at least three times with similar results. All attempts at replication were successful.
Randomization	The experimental grouping was performed randomly.
Blinding	The investigators and authors have been consistently blinded to the group allocation during data collection and analysis.

Reporting for specific materials, systems and methods

We require information from authors about some types of materials, experimental systems and methods used in many studies. Here, indicate whether each material, system or method listed is relevant to your study. If you are not sure if a list item applies to your research, read the appropriate section before selecting a response.

Materials & experimental systems

n/a	Involved in the study
☐	☒ Antibodies
☐	☒ Eukaryotic cell lines
☒	☐ Palaeontology and archaeology
☐	☒ Animals and other organisms
☒	☐ Clinical data
☒	☐ Dual use research of concern

Methods

n/a	Involved in the study
☒	☐ ChIP-seq
☒	☐ Flow cytometry
☐	☒ MRI-based neuroimaging

Antibodies

Antibodies used	Immunoblot analysis: IGFBP5 (Santa, sc-515116, 1:200), HER2 (Cell Signaling, #2165, 1:500), pHER2 Y1248 (R&D System, AF1768, 1:500), ROR1 (Cell Signaling, #16540, 1:500), pROR1 Tyr786 (Thermo Fisher, PA5-64807, 1:500), CREB 48H2 (Cell Signaling, #9197, 1:500), pCREB Ser133 (Cell Signaling, #9198, 1:500), IGF1R (Sangon Biotech, D155189, 1:1000), pIGF1R Tyr1165/1166 (Sangon Biotech, D155037, 1:1000), GAPDH (Cell signaling, #2118, 1:1000), beta-actin (Santa, sc-47778,1:1000).<br>Chromatin Immunoprecipitation: IgG (Abcam, ab27478), CREB (Cell Signaling, #9197).<br>Histology and Immunohistochemistry staining: pHER2 Y1248 (&D System, AF1768, 1:100), pROR1 Tyr786 (Thermo Fisher, PA5-64807, 1:100), pCREB Ser133 (Cell Signaling, #9198, 1:100), anti-GFP (abcam, 6556, 1:500), fluorochrome-conjugated Ab (Alexa 568, Invitrogen, #1011, 1:500).
Validation	Antibodies used were commercially available and were validated in multiple previous studies.<br>Anti mouse antibodies: IGFBP5, GAPDH, beta-actin.<br>Anti rabbit antibodies: HER2, pHER2 Y1248, ROR1, pROR1 Tyr786, CREB 48H2, pCREB Ser133, IGF1R, pIGF1R Tyr1165/1166.

Eukaryotic cell lines

Policy information about cell lines and Sex and Gender in Research

Cell line source(s)	GSC X01 (Generous gift from Myung-Jin Park, Korea Institute of Radiological & Medical Sciences, South Korea),<br>GSCs 448 and 131 (Generous gift from Do-Hyun Nam, Samsung Medical Center, South Korea),<br>GSC 83 (Generous gift from Ichiro Nakano, University of Alabama at Birmingham, United States),<br>GSC 772 (Patient-derived glioblastoma stem cell, National Cancer Center, South Korea).<br>293T cell was purchased from ATCC.
Authentication	We authenticated all GSCs by short tandem repeat (STR) analysis.
Mycoplasma contamination	We used Universal Mycoplasma Detection Kit to verify absence of mycoplasma contamination in our cell lines.
Commonly misidentified lines<br>(See ICLAC register)	No commonly misidentified cell lines were used in this study.

Animals and other research organisms

Policy information about studies involving animals; ARRIVE guidelines recommended for reporting animal research, and Sex and Gender in Research

Laboratory animals	5-week female BALB/c nude mice, provided by ORIENT BIO, Korea or SPF Biotechnology, China, Mice were group-housed in ventilated cages under controlled temperature and humidity with a 12-h light–dark cycle.
Wild animals	No wild animals were used in this research.
Reporting on sex	Female BALB/c nude mice were used in this research.
Field-collected samples	No field-collected samples were used in this research.
Ethics oversight	This animal studies were approved by the Institutional Animal Care and Use Committee of National Cancer Center, South Korea; and Animal Care and Use Committee of Laboratory Animal Center, Henan University, China.

Note that full information on the approval of the study protocol must also be provided in the manuscript.

Magnetic resonance imaging

Experimental design

Design type	Brian tumor volumes measurement by structural MRI.

Design specifications	The design specification of MRI is committed by week-wise interval for MRI trials. The MRI trial session is conducted every week.
Behavioral performance measures	N/A.

Acquisition

Imaging type(s)	T2-weighted images were acquired using a RARE sequence.
Field strength	Bruker Biospec 7T system (BioSpec 70/20 USR; Bruker, Germany) using mouse brain array coil.
Sequence & imaging parameters	Repetition Time (TR)=2500 ms; Echo Time (TE)=35 ms; Slice Thickness=0.7 mm; Echo Train Length=8; Number of Average (NEX)=4; Matrix size=256 x 192; Field of view (FOV)=20 x 20 mm.
Area of acquisition	Whole brain scan.

Diffusion MRI ☐ Used ☒ Not used

Preprocessing

Preprocessing software	Paravision 5.1
Normalization	No normalization defined MRI image in this research.
Normalization template	No normalization template was used in MRI imagine of this research.
Noise and artifact removal	No noise and artifact removal in MRI imagine of this research.
Volume censoring	No volume censoring was defined in MRI imagine of this research.

Statistical modeling & inference

Model type and settings	No statistical modeling was designed for MRI imagine of this research.
Effect(s) tested	No effects test was considered in MRI imagine of this research.

Specify type of analysis: ☒ Whole brain ☐ ROI-based ☐ Both

Statistic type for inference (See Eklund et al. 2016)	N/A.
Correction	No correction type was considered in MRI imagine of this research.

Models & analysis

n/a	Involved in the study
☒	☐ Functional and/or effective connectivity
☒	☐ Graph analysis
☒	☐ Multivariate modeling or predictive analysis
